# *Cannabis sativa* L. and Nonpsychoactive Cannabinoids: Their Chemistry and Role against Oxidative Stress, Inflammation, and Cancer

**DOI:** 10.1155/2018/1691428

**Published:** 2018-12-04

**Authors:** Federica Pellati, Vittoria Borgonetti, Virginia Brighenti, Marco Biagi, Stefania Benvenuti, Lorenzo Corsi

**Affiliations:** ^1^Department of Life Sciences, University of Modena and Reggio Emilia, Via G. Campi 103/287, 41125 Modena, Italy; ^2^Department of Physical Sciences, Earth and Environment, University of Siena, Strada Laterina 8, 53100 Siena, Italy

## Abstract

In the last decades, a lot of attention has been paid to the compounds present in medicinal* Cannabis sativa *L., such as Δ^9^-tetrahydrocannabinol (Δ^9^-THC) and cannabidiol (CBD), and their effects on inflammation and cancer-related pain. The National Cancer Institute (NCI) currently recognizes medicinal* C. sativa *as an effective treatment for providing relief in a number of symptoms associated with cancer, including pain, loss of appetite, nausea and vomiting, and anxiety. Several studies have described CBD as a multitarget molecule, acting as an adaptogen, and as a modulator, in different ways, depending on the type and location of disequilibrium both in the brain and in the body, mainly interacting with specific receptor proteins CB_1_ and CB_2_. CBD is present in both medicinal and fibre-type* C. sativa* plants, but, unlike Δ^9^-THC, it is completely nonpsychoactive. Fibre-type* C. sativa *(hemp) differs from medicinal* C. sativa*, since it contains only few levels of Δ^9^-THC and high levels of CBD and related nonpsychoactive compounds. In recent years, a number of preclinical researches have been focused on the role of CBD as an anticancer molecule, suggesting CBD (and CBD-like molecules present in the hemp extract) as a possible candidate for future clinical trials. CBD has been found to possess antioxidant activity in many studies, thus suggesting a possible role in the prevention of both neurodegenerative and cardiovascular diseases. In animal models, CBD has been shown to inhibit the progression of several cancer types. Moreover, it has been found that coadministration of CBD and Δ^9^-THC, followed by radiation therapy, causes an increase of autophagy and apoptosis in cancer cells. In addition, CBD is able to inhibit cell proliferation and to increase apoptosis in different types of cancer models. These activities seem to involve also alternative pathways, such as the interactions with TRPV and GRP55 receptor complexes. Moreover, the finding that the acidic precursor of CBD (cannabidiolic acid, CBDA) is able to inhibit the migration of breast cancer cells and to downregulate the proto-oncogene c-fos and the cyclooxygenase-2 (COX-2) highlights the possibility that CBDA might act on a common pathway of inflammation and cancer mechanisms, which might be responsible for its anticancer activity. In the light of all these findings, in this review we explore the effects and the molecular mechanisms of CBD on inflammation and cancer processes, highlighting also the role of minor cannabinoids and noncannabinoids constituents of Δ^9^-THC deprived hemp.

## 1. The Chemistry of* Cannabis sativa* L.

*Cannabis sativa* L. is a dioicous plant of the Cannabaceae family and it is widely distributed all over the world [1]. It has been used as a psychoactive drug, as a folk medicine ingredient, and as a source of textile fibre since ancient times [2]. The taxonomic classification of this plant has always been difficult, due to its genetic variability [1, 3]. Firstly, the genus* Cannabis* has been divided into three main species [1, 3, 4]: a fibre-type one, named* C. sativa *L., a drug-type one, characterised by high levels of the psychoactive compound Δ^9^-tetrahydrocannabinol (Δ^9^-THC), named* C. indica *Lam., and another one with intermediate properties, named* C. ruderalis *Janisch. Due the easy crossbreeding of these species to generate hybrids, a monotypic classification has been preferred, in which one species (*C. sativa*) is recognised and it is divided into different chemotypes [1, 3, 4]. On the basis of their cannabinoid profiles, five chemotypes have been recognised: chemotype I comprises drug type plants with a predominance of Δ^9^-THC-type cannabinoids; chemotypes III and IV are fibre-type plants containing high levels of nonpsychoactive cannabinoids and very low amounts of psychoactive ones; chemotype II comprises plants with intermediate characteristics between drug-type and fibre-type plants; chemotype V is composed of fibre-type plants which contains almost no cannabinoids [5].

For both medicinal and forensic purposes, the most important classification of* Cannabis* types is that into the drug-type and the fibre-type: the drug-type* Cannabis*, which is rich in psychoactive Δ^9^-THC, is used for medicinal or recreational purposes; the fibre-type* Cannabis*, rich of cannabidiol (CBD) or related compounds and almost devoid of Δ^9^-THC, is used for textile or food purposes [3]. Indeed, the well-known pharmacological activity of psychoactive cannabinoid Δ^9^-THC makes drug-type* Cannabis *one of the most investigated medicinal plants [3]. Fibre-type* Cannabis *(also known as hemp or industrial hemp) is at the moment underemployed for pharmacological purposes, while drug-type* C. sativa *is used in several diseases as a palliative therapy or in coadministration with primary therapy [1]. However, there has also been a growing interest in fibre-type* C. sativa* varieties in recent years [1], and those approved for commercial use by the European Community are 69 [5]. Many European countries have recognized the commercial value of hemp and a legal limit of 0.2-0.3%  Δ^9^-THC is usually applied [1].

**Figure 1:**
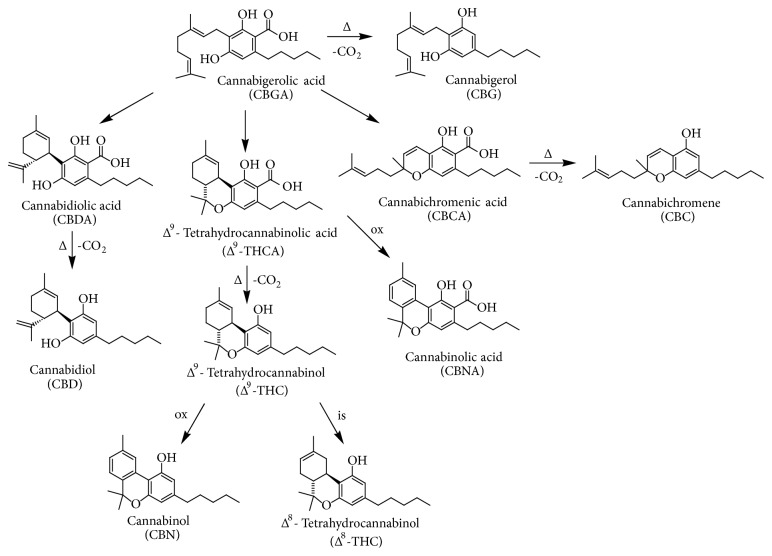
Chemical structures of main cannabinoids present in* Cannabis sativa* L. Abbreviation: Δ = heating; ox = oxidation; is = isomerization.

*C. sativa* is characterized by a complex chemical composition, including terpenes, carbohydrates, fatty acids and their esters, amides, amines, phytosterols, phenolic compounds, and the specific compounds of this plant, namely, the cannabinoids [2]. Cannabinoids are meroterpenoids (specifically C_21_ or C_22_ terpenophenolic compounds), obtained from the alkylation of an alkyl resorcinol with a monoterpene unit [3]. They are mainly synthesized in glandular trichomes, which are more abundant in female inflorescences [2]. More than 100 cannabinoids have been isolated, characterised, and divided into 11 chemical classes [4, 6]. Usually, the most abundant cannabinoids present in drug-type plants are Δ^9^-tetrahydrocannabinolic acid (Δ^9^-THCA) and Δ^9^-THC, while fibre-type plants are known to contain mainly cannabinoic acids, such as cannabidiolic acid (CBDA) and cannabigerolic acid (CBGA), followed by their decarboxylated forms, namely, cannabidiol (CBD) and cannabigerol (CBG) (Figure 1) [7, 8]. Other minor cannabinoids include cannabichromenic acid (CBCA), cannabichromene (CBC), cannabinolic acid (CBNA), and cannabinol (CBN), with the last two being the oxidative degradation products of Δ^9^-THCA and Δ^9^-THC, respectively, present in aged* Cannabis* (Figure 1) [1, 3, 4, 7–10]. Δ^9^-THC can also be transformed by isomerization to Δ^8^-THC (Figure 1), which is an artefact. It should be pointed out that cannabinoids are biosynthesized in the acid form in plant tissues; then, they can generate their decarboxylated counterparts under the action of heat and light, by means of a spontaneous decarboxylation [1, 3, 4, 7–10].

Many of the psychoactive effects of Δ^9^-THC are mediated by CB_1_ receptors, while nonpsychoactive cannabinoids, such as CBD, have low affinity for both CB_1_ and CB_2_ receptors [3]. The interaction with CB_1_ receptors is responsible for the analgesic effect of Δ^9^-THC, due to their role in the transmission of the nociceptive information in various tissues [3]. CB_2_ receptors are highly expressed in some cells of the immune system and they are believed to have a role in the immune cell function, thus explaining the immunomodulatory properties of Δ^9^-THC. CB_2_ receptors are also considered to be involved in neuroinflammation, atherosclerosis, and bone remodelling [3].

In the ambit of nonpsychoactive compounds, CBD represents the most valuable one from the pharmaceutical point of view, since it has been found to possess a high antioxidant and anti-inflammatory activity, together with antibiotic, neuroprotective, anxiolytic, and anticonvulsant properties [1, 3, 11–14]. CBDA has antimicrobial and antinausea properties [1, 11, 13], while CBG has anti-inflammatory, antimicrobial, and analgesic activities [1, 11, 13, 15]. Thanks to its lack of psychoactivity, CBD is one of the most interesting compounds, with many reported pharmacological effects in various models of pathologies, from inflammatory and neurodegenerative diseases, to epilepsy, autoimmune disorders like multiple sclerosis, arthritis, schizophrenia, and cancer [16]. In the presence of Δ^9^-THC, CBD is able to antagonize CB_1_ at low concentration; this supports its regulatory properties on Δ^9^-THC adverse effects like tachycardia, anxiety, sedation, and hunger in animals and humans [16]. CBD has also been found to be a negative allosteric modulator of the CB_1_ receptors and an inverse agonist of CB_2_ receptors, the second activity partly explaining its anti-inflammatory activity [16]. Different targets have been described in the literature for nonpsychoactive cannabinoids, including the transient potential vanilloid receptor type-1 (TPVR-1) channels, the peroxisome proliferator-activated receptor *γ* (PPAR*γ*) GPR55, the 5-hydroxytryptamine receptor subtype 1A (5-HT1A), glycine *α*1 and *α*1*β* receptors, the adenosine membrane transporter phospholipase A2, lipoxygenase (LO) and cyclooxygenase-2 (COX-2) enzymes, and Ca^2+^ homeostasis [11, 16].

Concerning other phenolics present in* C. sativa*, several flavonoids have been identified, belonging mainly to flavones and flavonols, together with cannflavins A and B, which are* C. sativa* typical methylated isoprenoid flavones [17].* Cannabis *flavonoids exert several biological effects, including properties possessed also by cannabinoids and terpenes [2]. Anti-inflammatory, neuroprotective, and anti-cancer activities have been described for these compounds [2]. In particular, cannflavin A and B are known to possess an anti-inflammatory action [2]. Microsomal prostaglandin E_2_ synthase (mPGES-1) and 5-LO have been identified as the molecular targets of cannflavins A and B [18]. An antimicrobial and antileishmanial activity has also been demonstrated for cannflavin B [17]. Cannflavin A has shown a good antileishmanial activity and a moderate antioxidant action [17]. In the ambit of* Cannabis* phenolics, canniprene, which is a dyhydrostilbene unique to* C. sativa*, represents an interesting compound [19]. If compared with cannflavin A, which is the most potent cannflavin, canniprene has been found to be superior at inhibiting 5-LO, but it is less effective for mPGES-1 inhibition [19].

As regards the other compounds present in* C. sativa*, terpenes are responsible for the characteristic scent of the plant. Both mono- and sesquiterpenes have been detected in roots and aerial parts of* Cannabis* and they are mainly produced in secretory glandular hairs [2]. In the ambit of monoterpenes, *β*-myrcene is known to possess anti-inflammatory, analgesic, and anxiolytic properties [2]. As for sesquiterpenes, *β*-caryophyllene has anti-inflammatory and gastric cytoprotector activities; it is also able to bind to the CB_2_ receptors and, in this context, it is considered as a phytocannabinoid [2].

Several interactions between* Cannabis* secondary metabolites have been described in the literature [2]. In addition to the capacity of CBD to reduce Δ^9^-THC side effects, terpenes are able to increase blood-brain barrier permeability, thus affecting Δ^9^-THC pharmacokinetics; they can also influence the affinity of Δ^9^-THC for CB_1_ receptors and interact with neurotransmitter receptors, thus contributing to cannabinoid-mediated analgesic and psychotic effects [2]. Finally, also flavonoids may modulate the pharmacokinetics of Δ^9^-THC, by means of the inhibition of hepatic P450 enzymes (3A11 and 3A4) [2].

### 1.1. Cannabidiol (CBD)

Many studies have expanded the concept that inflammation is a critical component of tumour progression [20]. Indeed, several cancers originate from infection, chronic irritation, and inflammation [20]. Tumour microenvironment, which is largely regulated by inflammatory cells, displays a key role in the neoplastic process, fostering proliferation, survival, and migration [20]. In addition, cancer cells have co-opted some of the signalling molecules of the innate immune system for invasion, migration, and metastasis [20].

By focusing the attention on hemp nonpsychoactive cannabinoids, CBD has been demonstrated to be useful in the treatment of different inflammatory ailments, including bowel diseases (e.g., Crohn's and ulcerative colitis), neuronal diseases (e.g., Parkinson and Alzheimer), and a wide range of inflammatory skin diseases (e.g., atopic dermatitis and psoriasis) [21].

As regards cancer, CBD has exhibited antiproliferative and proapoptotic activities, thus demonstrating modulating the tumorigenesis in different types of cancer, including breast, lung, colon, brain, and others [21].

In this context, this review is focused on the effects and the molecular mechanisms of CBD and related compounds on inflammation and cancer processes, highlighting also the role of other related nonpsychoactive cannabinoids and noncannabinoids constituents of fibre-type hemp. Although it has been reported that CBD is able to bind several protein complexes, such as PPAR*γ* and 5HT1, their role in CBD-mediated anticancer activity is still poor documented. For this reason, the attention is focused mainly on the interaction between CBD and three putative molecular targets such CB_2_, GPR55, and TRPV1/2 protein receptors, where there is an extensive literature and several molecular mechanisms have been proposed.

## 2. The Role of the Endocannabinoid System in Peripheral Inflammation

Endocannabinoids and their metabolic enzymes and receptors have been identified in monocytes, macrophages, basophils, lymphocytes, and dendritic cells. In these cells their role is to modulate immune function in an autocrine and paracrine way [22].

In human peripheral blood cells, CB_1_ are expressed by B cells, NK cells, neutrophils, CD8+ T cells, monocytes, and CD4+ T cells, in a decreasing rank order, whereas CB_2_ mRNA is expressed by human B cells, NK cells, monocytes, neutrophils, and T cells, in a decreasing rank order [23].

CB_2_ expression in human B cells increases after the activation by anti-CD40 antibody. However, differentiation of B cells is accompanied by decreased expression of CB_2_. CB_2_ levels in macrophages undergo changes correlated with cell activation or with inflammation. Indeed, macrophages express higher levels of CB_2_; so, the functions of macrophages in these states of activation may be the most sensitive to the actions of cannabinoids. These data suggest a physiological role of the endocannabinoid system in the functions of immune cells with respect to inflammation [24].

Both 2-arachidonylglycerol (2-AG) and anandamide (AEA) play an immunomodulatory role through their activity on CB_2_. CB_2_ activation typically inhibits the functions of immune cells with intracellular signaling mechanisms, including the inhibition of adenylate cyclase activity by Gi/o proteins and activation of MAPKs. Indeed, CB_2_ are able to inhibit the production of proinflammatory cytokines, like TNF-*α*, IL-6, and IL-8 in human monocytes and macrophages, and to reduce the release of TNF-*α*, IL-2, and IFN-*γ* in activated human peripheral lymphocytes.

Moreover, a relationship between the endocannabinoid system and toll-like receptors (TLR) has been reported, with TLR activation enhancing the production of endocannabinoids and cannabinoids suppressing TLR-induced inflammatory response [25].

### 2.1. Nonpsychoactive Cannabinoids and Peripheral Inflammation

**Figure 2:**
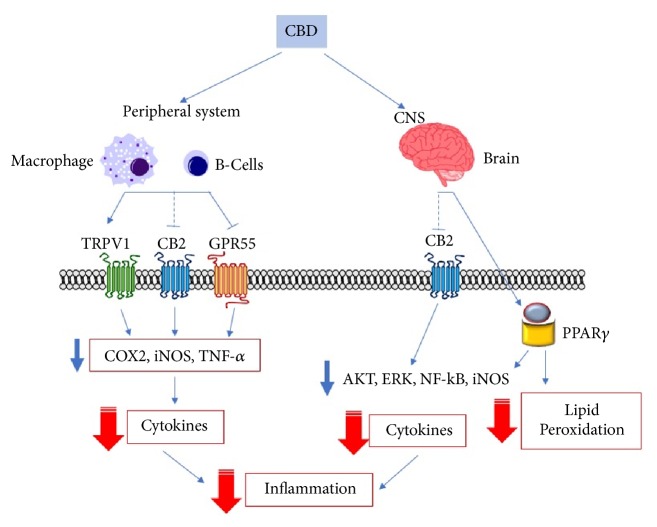
General representation of the signaling pathways involved in CBD anti-inflammatory effects. Cannabinoids reduce peripheral inflammation by acting at TRPV1, CB_2_, and GPR55 receptors; these interactions lead to downregulation of enzymes involved in the production of prostaglandins, reactive oxygen species, and cytokines. MAPK inhibition and NF-kB downregulation, together with PPAR*γ*-mediated reduction of lipid peroxidation, are also involved in the anti-inflammatory effects of cannabinoids in the CNS. Abbreviations: CBD, cannabidiol; CNS, central nervous system, CB_2_, cannabinoid receptor 2; TRPV1, receptor potential channel subfamily V member 1; GPR55, orphan G-protein coupled receptor 55; Akt, protein kinase B; ERK, extracellular signal-regulated kinases; NF-kB nuclear factor kappa-light-chain-enhancer of activated B cells; iNOS, inducible nitric oxide synthase; COX2, cyclooxygenase 2; TNF-*α*, tumor necrosis factor alpha; PPAR*γ*, peroxisome proliferator-activated receptor gamma.

The study of the anti-inflammatory effects of cannabinoids from* C. sativa* L. is of current interest [26, 27]. Although Δ^9^-THC has been reported to possess anti-inflammatory in a plethora of* in vitro* and* in vivo* models [28–38], a number of reports have highlighted the role of nonpsychoactive cannabinoids in inflammatory processes (Figure 2).

CBD anti-inflammatory effect may be mediated by cannabinoid receptors (CBr), adenosine A2A receptors, TRPV1 receptors, GPR55 receptors, and CB2/5HT(1A) heterodimerization [27].* In vivo*, CBD has been able to reduce inflammation in a murine model of colitis, even if Δ^9^-THC was more effective [28]. In a carrageenan-induced inflammation model in rats, CBD reduced PGE_2_, nitric oxide (NO), and malondialdehyde production, together with COX activity [39]. CBDA has been found to possess a dual inhibitory effect on COX, through downregulation [40] and enzyme inhibition [35]. CBD has also completely inhibited the production of TNF-*α* in LPS-stimulated RAW264.7 macrophages [41]. Moreover, a reduction of IL-1*β* and TNF-*α* levels has been observed in mitogen-activated human PBMC [42]. More recently, CBD has been found to significantly reduce cytokines production in an* in vitro *model of allergic contact dermatitis, using HaCaT cells [43].

The ability to activate and desensitize TRPV4 channels is linked to the reduction of NO production exerted by CBG in LPS-stimulated macrophages [44]. Moreover, CBG and CBGA resulted in inhibiting COX activity, even at high micromolar concentrations [35].

CBC has reduced nitrites production, IL-10 and IFN-*γ* levels in murine macrophages, without influencing CBr [25]. Moreover, CBC has decreased intestinal hypermotility in mice, in a manner not dependent on CBr and TRPA1 receptors [45].

Concerning the effect of other* C. sativa* constituents, cannflavins anti-inflammatory activity has been poorly investigated, but it seems to be related to the reduction of PGE_2_ and the inhibition of 5-lipoxygenase [18, 46].

As regards terpenes, myrcene and limonene are able to reduce cytokines production and inhibit NF-*κ*B and MAPK in LPS-stimulated murine macrophages [47]. *β*-Caryophyllene reduced TNF-*α* and IL-1*β* production by downregulating MAPK and reducing ERK phosphorylation in LPS-stimulated PBMC [48].

As far as peripheral inflammation is concerned,* C. sativa *has been used medicinally for centuries to treat a variety of disorders, including those associated with the gastrointestinal tract. Recent investigations have highlighted the involvement of the endocannabinoid system in the physiology of the gastrointestinal function and its possible deregulation in gastrointestinal pathology [49]. The precise mechanisms across tissue departments that are under the regulatory control of the endocannabinoid system have not been fully understood [49].

Cannabinoids have been found to modulate intestinal permeability in an* in vitro* model. Both Δ^9^-THC and CBD are able to restore the increased permeability induced by either EDTA or endocannabinoids whether applied to the apical or basolateral membrane of Caco-2 cells [50]. These data suggest that endocannabinoids may play a role in the modulation of gut permeability and that* Cannabis*-based medicines may possess therapeutic benefit in a variety of gastrointestinal diseases characterized by abnormal intestinal permeability, such as inflammatory bowel disease (IBD) and shock [50].

These findings have been further confirmed in another* in vitro *model of intestinal inflammation. In particular, endocannabinoids caused further increases in Caco-2 cell permeability in the presence of cytokines, whereas both Δ^9^-THC and CBD restored increased permeability induced by cytokines [51]. The effects of cytokines on increased permeability were inhibited by a CB_1_ receptor antagonist and a 2-AG synthesis inhibitor and were enhanced by inhibitors of the degradation of AEA or 2-AG, suggesting that local production of endocannabinoids activating CB_1_ may play a role in the modulation of gut permeability during inflammation [51].

CBD anti-inflammatory effects on the acutely inflamed human colon have also been investigated in combination with palmitoylethanolamide (PEA) in cultured cell lines and this effect was compared with experimentally inflamed explant human colonic tissue [52]. In particular, Caco-2 cells and human colonic explants collected from elective bowel cancer, inflammatory bowel disease (IBD), or acute appendicitis resections were used. CBD and PEA were able to prevent cytokine production in human colonic explant tissue via PPAR*α*, CB_2_, and TRPV1, but not in Caco-2 cells [52]. These effects extend into chronic inflammatory processes, such as IBD, but also acute inflammatory conditions, such as appendicitis. Since these two compounds are well tolerated in humans with few side effects, their clinical use in treating IDB can be very useful [52].

In another study, CBD has been demonstrated to improve* Clostridium difficile* toxin A-induced damage in Caco-2 cells, by inhibiting the apoptotic process and restoring the intestinal barrier integrity, through the involvement of CB_1_ receptors [53].* Clostridium difficile* infection is the leading cause of hospital-acquired diarrhea and pseudomembranous colitis.* Clostridium difficile* toxin A significantly affects enterocytes permeability leading to apoptosis and colonic mucosal damage. Given the absence of any significant toxic effect in humans, CBD may ideally represent an effective adjuvant treatment for* Clostridium difficile*-associated colitis [53].

In addition to the protective role of* Cannabis *components on the inflamed intestine, an additional positive aspect is their potential role in preventing imbalances of gut microbiota. This aspect not only is relevant for the treatment of several gastrointestinal disorders, such as IBD and obesity, but also has implications for the treatment of colorectal cancer (CRC). The impact of the endocannabinoid system on gut microbiota is a relatively new and emerging field wherein the interplay between cannabinoids and metabolic syndrome has been the focus so far. Recent data have suggested that Δ^9^-THC prevents further exacerbation of the Firmicutes:Bacteroidetes ratio typically found in obesity, resulting in weight-loss, indicating that* Cannabis* may play a role in CRC prevention as well [54]. Further studies are needed to determine whether CBD has the same effect on gut microbiota with respect to the balance of Firmicutes:Bacteroidetes to evaluate its application in halting the progression of the obese microbiota profile present in CRC, with the hopes of delaying this disease onset [54].

## 3. The Role of the Endocannabinoid System in Neuroinflammation

CB_1_ receptors are much more expressed in the brain if compared to CB_2_ [55]. However, CB_2_ can be upregulated under neuroinflammatory conditions and as a result of the invasion of peripheral cells expressing CB_2_ [56].

The neuroprotective effect of endocannabinoids involves the suppression of proinflammatory cytokines and the increase of anti-inflammatory cytokines production. This altered expression is mainly mediated by the activation of the MAPKs pathway and regulated primarily by MKP-1 [23].

A decrease of TNF-*α*, IL-6, IL-1*β*, and IL-12 levels in rats brain has been observed after treatment with LPS in several studies. Nevertheless, cannabinoids have been found to increase the production of cytokines, including TNF-*α*, IL-6, IL-1*β*, and IL-10, when administered alone [57].

Cytokines may regulate the normal activity of the endocannabinoid system in different ways: for example, IL-4 and IL-10 are able to stimulate FAAH activity, whereas IFN-*γ* and IL-12 decrease FAAH expression, resulting in an increase of AEA levels [58, 59]. TNF-*α* and IL-6 are the major cytokines which can regulate CBr activity. Indeed, these cytokines have pro- and anti-inflammatory properties, depending on a variety of factors. Recent studies have shown that TNF-*α* provides a crucial signal for stem cells migration through CB_1_/CB_2_ signaling. The activation of TNF-*α* receptor then leads to 2AG synthesis, which may act on CB_1_ and CB_2_. This activity leads to a promotion of stem cells proliferation and migration that might have important implications for brain self-repairing processes [60].

The cannabinoid system and cytokine network are directly related. CB_1_ and CB_2_ expression is significantly induced by the presence of TNF-*α*. This occurs, at least in part, through the activation of NF-*κ*B, which could be induced by stimulation of TNF receptor. Upon activation, NF-*κ*B translocates into the nucleus, where it binds DNA and triggers the transcription of target genes, some of which encode inflammatory proteins and may include the CBr genes [61].

### 3.1. Nonpsychoactive Cannabinoids and Neuroinflammation

Evidences suggest that controlled neuroinflammation is crucial for tissue repair within the brain [62, 63]. However, prolonged exposure to inflammatory conditions in the brain has been linked with the development of neurodegenerative diseases, such as the Alzheimer's and Parkinson's diseases, and multiple sclerosis [64]. In Alzheimer's disease, misfolded and aggregated proteins are recognized by microglia and activate an innate immune response characterized by the release of inflammatory mediators, contributing to the disease progression and severity [65]. The role of neuroinflammation in the pathogenesis of Parkinson's diseases is supported by several experimental evidences, even if it remains unclear whether the inflammatory processes are involved in the initiation of the disease or are secondary consequences of the brain injury [66, 67]. Regarding multiple sclerosis, inflammation appears to be mediated by T-helper 1 cells, with enhanced presence of Th1/Th17 cells being found in central nervous system (CNS) tissue, cerebrospinal fluid (CSF), and blood of patients [68, 69].

*C. sativa* and its constituents have been reported to be promising candidates for the management of several neuroinflammatory conditions (Figure 2) [70]. CBD, similarly to Δ^9^-THC, has been able to reduce neurotoxicity in SH-SY5Y neuronal cells exposed to LPS-conditioned BV2 microglial cells medium, by modulating BV2 morphological plasticity and cytokines signaling through the activation of GPR18 receptors [71].

In an* in vitro* model of neuroinflammation using LPS-stimulated rat microglia, CBD has suppressed TNF-*α*, IL-1*β*, and IL-6 release, by reducing NF-*κ*B phosphorylation, together with COX and iNOS activation, in a CB_2_ dependent manner [72, 73]. Interestingly, CBD has caused a downregulation of Akt and ERK pathways in human glioma cells [74]. The inhibition of ATP-induced intracellular calcium increase, together with the inhibition of NO production, has been suggested as a mechanism by which CBD can reduce microglia activation [75]. In cultured rat primary astrocytes, CBD has reduced the A*β*-induced release of NO, IL-1*β*, and TNF-*α*, by activating PPAR*γ* and inhibiting NF-*κ*B nuclear translocation [76]. In another work, CBD has also inhibited the neurotoxic effects of protease-resistant prion protein (PrPres) and it has affected PrPres-induced microglial cell migration in a concentration-dependent manner; so, it may protect neurons against the multiple molecular and cellular factors involved in the different steps of the neurodegenerative process, which takes place during prion infection [77]. More recently, the neuroprotection of fibre-type hemp extracts and CBD was assessed in human neuroblastoma SH-SY5Y and microglial BV-2 cell lines in the presence of rotenone as the toxic agent, also in serum-free conditions [78]. The decarboxylated hemp extract has shown a mild neuroprotective activity on BV-2 cells treated with rotenone, higher than that of pure CBD [78]. As regards serum-free experiments, the nondecarboxylated hemp extract was the most effective neuroprotective agent toward SH-SY5Y cells, while BV-2 cells were better protected from the toxic insult by the decarboxylated extract and CBD [78].

Concerning other cannabinoids, the anti-inflammatory properties of CBG have been described in an* in vitro* model of neuroinflammation, using NSC motor neurons conditioned with the medium of LPS-stimulated murine macrophages. CBG treatment in macrophages has prevented neuronal cytotoxicity by reducing inflammation, (i.e., IL-1*β*, TNF-*α*, and IFN-*γ* production, together with PPAR*μ* protein levels) and oxidative stress, reducing nitrotyrosine, SOD1, and iNOS protein levels and restoring Nrf-2 levels [79].

As regards other* C. sativa* components, *β*-caryophyllene is able to reduce the production of IL-1*β*, TNF-*α*, IL-6, and ROS, through the inhibition of NF-kB nuclear translocation in murine microglial cells, after hypoxic exposure [80].

In the ambit of CNS pathology and, in particular, regarding Alzheimer's disease, studies in rodents have demonstrated the ability of CBD to reduce reactive gliosis and neuroinflammatory response as well as to promote neurogenesis [81]. Moreover, in an* in vitro* model using SH-SY5Y human neuroblastoma cells, CBD has been able to induce ubiquitination of APP protein, reducing *β*-amyloid peptide production and neuronal apoptosis through activation of PPAR***γ*** [82]. These results are consistent with those obtained by Hughes and coworkers, who have observed a PPAR*γ* mediated neuroprotective effect of CBD in the hippocampus of C57Bl/6 mice [83].

## 4. Inflammation and Cancer

Cancer is the second leading cause of death worldwide, and it accounts for about 8.8 million deaths in 2015 (GHO 2018 data); nearly 1 of 6 deaths is due to cancer. Cancer is a multistep disease characterized by a formation of a preneoplastic lesion (initiation processes) which, by time, progresses into malignant tumor. Generally, cell transformation is a combination of intrinsic genetic factors and external exposure to physical, chemical, and biological carcinogens. However, it must be underlined that ageing and life style are others fundamental factors for the development of the disease. Indeed, the incidence of cancer rises dramatically with age, probably due to the decreased efficacy of cellular repair mechanisms, while tobacco, alcohol, unhealthy diet, and physical inactivity are the major global cancer risks. A number of evidences pointed out that chronic inflammation, independently of the triggering agent, could be responsible of almost 20% of human cancers [84]. As described above, inflammation* per se* is not dangerous, since it protects the body by increasing host defense and it is self-limiting. However, persistent and deregulated inflammation is associated with an increased risk of malignant diseases [85]. Cells and mediators of the innate immune system have been detected in many cancers, even when inflammation is not implicated in tumor development [85, 86]. This finding suggests that inflammatory conditions and carcinogenesis might share common pathways, such as proliferation, increased cell survival, and migration, where cytokines and growth factors play a pivotal and fundamental role. Therefore, not only can inflammation cause cancer, but also cancer causes inflammation [87]. Thus, in the tumor microenvironment, inflammatory mediators regulate a number of proinflammatory responses, acting in an autocrine and/or paracrine manner, leading to either an antiproliferative response or an increase of cancer promotion through the inhibition of protective immune response [88]. In this context, it has been shown that the activation of the proinflammatory NF-kB pathway has a tumor prosurvival effect, giving chemotherapy resistance to cancer cells in an Akt-independent pathway, but involving the epidermal growth factor (EGF) activating signaling [89]. This interesting link between inflammation and growth factors, such as EGF/EGFR, configures an intriguing perspective in the study of the possible correlation between inflammatory processes and aberrant cell growth. Studies carried out on liver cancer have shown that chronic tissue damage and inflammation in liver result in a sustained overexpression and overstimulation of the EGFR pathway and that the deregulated EGFR signaling has been reported to play an important role in the development of liver cancer [89]. Proinflammatory stimuli activated by EGFR promote the release of EGFR ligands, such as heparin-binding-EGF (HB-EGF), from liver cancer cells and endothelial cells, which stimulate the proliferation of initiated hepatocytes [89], and further potentiate their aggressive behavior [90]. Moreover, the finding that CBD suppresses the activation of EGF/EGFR signaling transduction pathway and its downstream targets Akt, ERK, and NF-kB suggests that the effect of* C. sativa* extract might play an important in the modulation on the intricate relationship between growth factors, inflammation, and cell growth [90]. Indeed, the ability to inhibit proinflammatory pathways, as described in the previous chapter, strongly indicates that cannabinoids are antiproliferative compounds by possibly interfering with NF-kB/EGF/EGFR pathway. This hypothesis has been further supported by Elbaz et al. [91], who have demonstrated that CBD, acting on its receptors, changes cytokine secretion, such as CCL3, GM-CSF, and MIP-2 proteins, from 4T1.2 tumor cells compared to vehicle-treated cells, thus decreasing the recruitment of macrophages to the tumor microenvironment and, therefore, suppressing both angiogenesis and the invasive potential of cancer cells. In addition, the presence of GPR55 receptor, which is able to bind CBD, on NK cells, represents a possible novel modulatory activity of NK cell responsiveness [92]. Noteworthy, the noncanonical cannabinoid receptor G coupled receptor GPR55-mediated NK cell stimulation and/or inhibition is of particular importance in tumor immune-surveillance, since these cells play a pivotal role in the recognition and elimination of malignant cells.

## 5. The Role of the Endocannabinoid System in Cancer

**Figure 3:**
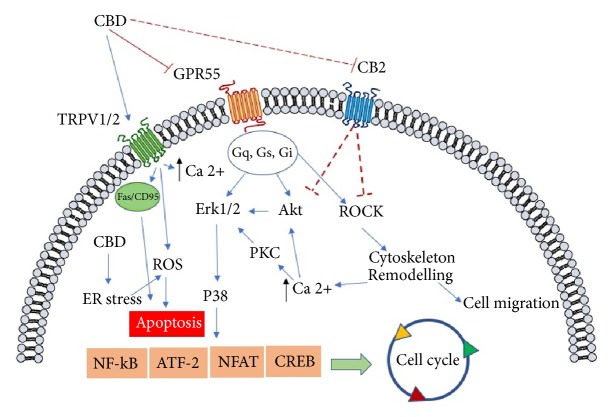
General representation of the signaling pathways involved in CBD anticancer mediated effects. Cannabinoid-induced apoptosis relies on the stimulation of endoplasmic reticular (ER) stress and through stimulation of TRPV channel. The signaling route involving the arrest of cell proliferation is mediated by the antagonism mainly on GPR55, which causes an inhibition of the activation of ERK pathway; in addition, the block of ROCK activation might be responsible for the antimigratory effect elicited by cannabidiol. CBD, cannabidiol; CB_2_, cannabinoid receptor 2; TRPV1/2, receptor potential channel subfamily V members 1 and 2; GPR55, orphan G-protein coupled receptor 55; ROS, reactive oxygen species; ER, endoplasmic reticulum; p8, protein p8 (Nuclear Protein 1, NUPR1); CHOP, CCAAT/-enhancer-binding protein homologous protein; ATF2, activating transcription factor 2; CREB, cAMP response element-binding protein; Akt, protein kinase B; ROCK Rho-associated protein kinase; NFAT, nuclear factor of activated T-cells; NF-kB, nuclear factor kappa-light-chain-enhancer of activated B cells; PKC, protein kinase C; P38, mitogen-activated protein kinases.

The role of the endocannabinoid system in cancer biology is a controversial matter. Indeed, if on one hand an increase, although with a different pattern and extent, of endocannabinoid receptors CB_1_ and CB_2_ in various types of cancers has been observed, on the other hand the endocannabinoid system seems to play a tumor suppressing role on colon carcinoma in a genetic modified mouse model, carrying a knockdown of CB_1_ gene [93]. However, the majority of researches have reported an increase of CB_1_ and CB_2_ in different types of cancer. In particular, CB_1_ receptor has been found to be upregulated in cellular hepatocarcinoma [94] and in Hodgkin lymphoma cells [95], and its expression correlates with the severity of the disease in human epithelial ovarian cancer [96], whereas CB_2_ has been found to be overexpressed in human breast adenocarcinomas associated with HER2+ [97] and in glioma [98]. Moreover, CB_1_ and CB_2_ expression has been proposed to be a factor of bad prognosis following surgery in stage IV of colorectal cancer [99]. All these findings support the hypothesis that cannabinoids might interfere with cancer biology, acting on CB_1_ and CB_2_ receptors in a wide range of cancer types, in particular for Δ^9^-tetrahydrocannabivarin (Δ^9^-THCV), which is a homologue of Δ^9^-THC with a propyl side chain instead of a pentyl group. However, since nonpsychoactive cannabinoids, such as CBD, do not bind with high affinity to both CB_1_ and CB_2_, alternative pathways should be considered in order to analyze the molecular mechanisms of CBD anticancer activity (Figure 3).

### 5.1. Nonpsychoactive Cannabinoids and Cancer

In cancer treatment, cannabinoids, such as dronabinol (synthetic Δ^9^-THC) and nabilone (a synthetic cannabinoid similar to Δ^9^-THC), are mainly used in association with chemotherapy in order to decrease its side effects such as pain, weight loss, nausea, and vomiting, although their use is still limited due to their psychoactive side effects [100]. However, incoming evidences have suggested that their activity could not be ascribed solely to these “palliative” effects, but rather the compounds could possess some interesting properties in terms of inhibition of tumor cell proliferation.

The first evidence of the ability of cannabinoids, and in particular Δ^9^- and Δ^8^-THC (Figure 1), to reduce the growth of lung adenocarcinoma both* in vitro* and* in vivo* has been reported by Munson et al. in 1975 [101]. As mentioned above, in recent years a number of researches have been made to evaluate the antiproliferative and proapoptotic effects of cannabinoids in both* in vitro* and* in vivo* models and in different cancer types, such as glioma, breast, pancreas, prostate, colorectal and lung carcinoma, and lymphoma [102–109]. These results prompted up the research of the possible molecular mechanism involved in the effects mediated by cannabinoids, together with the discovery of new activities elicited by these compounds, such as the interference with angiogenesis, cancer cell migration, and invasion [110]. All these findings strongly reinforce the idea that these compounds are able to control the cell survival/death fate and, therefore, they could be good candidates in cancer therapies.

### 5.2. Nonpsychoactive Cannabinoids and GPR55

GPR55 is a, so-called, orphan receptor protein, constituted by 319 amino acids, and it is present in the chromosome 2q37 [111]. GPR55 has been identified for the first time in 1999, and it belongs to the *δ* group of rhodopsin-like G protein coupled receptors (GPCRs) [112]. GPR55 possesses different biological functions based upon its localization: it controls the motility of the gastrointestinal tract, angiogenesis, and neuropathic pain; it modulates the inflammatory processes and it is involved in intracellular signaling involving the upstream signaling of RhoA, ROCK, ERK, and p38 mitogen activated protein kinase pathways, and Ca^2+^ release, which in turn modulate the downstream, cell motility and stiffness and the transcription factors nuclear factor activated T cell (NFAT), nuclear-factor-kB (NF-kB), cAMP response elements binding protein (CREB), and the activating transcription factor-2 (ATF2) [113–115]. The modulation of these important biological determinants indicates that GPR55 is a possible pharmacological target in a number of diseases where these pathways are deregulated, such as cancer. Increased expression of GPR55 and severity and malignancy of the disease has been reported in different cancer types, such as the human pancreatic ductal adenocarcinoma, the squamous cell carcinomas human astrocytoma, the melanoma, the B lymphoblastoma [116–118], and the hepatocellular carcinoma as well [94]. Although the pharmacology of GPR55 remains controversial, a number of evidences have suggested that it is a non-CB_1_/CB_2_ receptor able to bind nonpsychoactive cannabinoids and CBD in particular is its putative ligand, acting as an antagonist [119, 120]. In this view, Shrivastava et al. [121] have demonstrated that CBD is able to kill breast cancer cells by inducing ER stress, enhancing ROS generation and inhibiting mTOR signaling. In addition, CBD has been found to regulate the balance between autophagy and mitochondria-mediated apoptosis. This latter effect could be mediated by the antagonistic effect of CBD to GPR55, since its inhibition with anandamide allows the recruitment of the death receptor Fas into cell membrane through the activation of protein G*α*12 and Jun N-terminal kinase [113]. Moreover, Solinas et al. [74] have shown the antiproliferative and anti-invasive effects of CBD in U87-MG cells in a CB_1_/_2_ independent manner, and these effects have been extended to T98G glioma cells, a Δ^9^-THC-resistant lineage, thus suggesting a possible alternative pathway from that involving CB_1_/CB_2_ receptors. CBD effects are mediated by a significant downregulation of ERK and PI3K/Akt kinases, which are fundamental for cell survival and proliferation (Figure 3). The finding that stable overexpression of this GPR55 in HEK293 cells led to increased levels of phosphorylated extracellular signal-regulated kinase (ERK) [117] involved in cell proliferation strongly indicates that GPR55 is a target for CBD-mediated anticancer activity. Moreover, the fact that CBD downregulates the expression of angiogenic related proteins both* in vitro* and* in vivo*, such as matrix metallopeptidase 9 (MMP9), tissue inhibitor of metalloproteinases 1 (TIMP1), serpinE1-plasminogen activator inhibitor type-1 (PAI-1), CXCL16, ET-1, PDGF-AA, and IL-8 [74], reinforces the hypothesis that CBD might exert its antiangiogenic activity through the interaction with GPR55. Indeed, a clear relationship between GPR55 and angiogenesis has been reported by Zhang et al. [122], where the endothelial vascular cells regulate GPR55-mediated angiogenesis through the autocrine release of LPI; once GPR55 was downregulated in primary human microvascular endothelial cells, a decrease in angiogenesis was observed. Even if all these data clearly indicate that the antagonism of CBD on GPR55 activity inhibits cancer cell proliferation and angiogenesis and increases apoptosis, further studies are necessary, in order to better understand and to complete the portrait of the relation between CBD, GPR55, and cancer biology both in* in vitro* and* in vivo* models. Moreover, other protein receptors may be able to bind CBD, and in turn they can be of importance in the modulation of cancer growth.

### 5.3. Cannabinoids and TRPVs

Beside the discussed GPR55 receptors for nonpsychoactive cannabinoids, another non-CB_1_/CB_2_ receptor system, i.e., the transient receptor protein of vanilloid types 1 and 2 (TRPV1, TRPV2), has been proposed to bind either endocannabinoid or phytocannabinoids. The TRP receptors control mainly body temperature perception, thermal pain, and noxious stimuli and they are involved in several biological functions, such as cell proliferation [123].

In particular, the TRPV vanilloid channels belong to a superfamily of channels called “Transient Receptor Potential” (TRP), which promote calcium entry into the cells. The most extensively studied receptor in the ambit of the TRPV family is represented by TRPV1. TRPV1 and TRPV2 are ubiquitously expressed throughout the body, with a particular abundance in the central nervous system (CNS), and they differ both in the activities that they mediate and in their pharmacological profiles [124]. TRPV1 is activated by heat and, once activated, it allows the entrance of calcium and magnesium into the cells. Upon activation, the channel undergoes a rapid desensitization in a Ca-dependent manner.

TRPV1 is modulated by a number of bioactive compounds, such as capsaicin, piperine, camphor, CBD, and the endocannabinoid anandamide, which activate the channel. TRPV2 is not modulated by pungent-tasting compounds, such as capsaicin or piperine, but it shares with TRPV1 the activation elicited by CBD, related cannabinoids, and probenecid. It differs also from TRPV1 for its role in various osmo- or mechanosensory activities rather than noxious heat stimuli [125]. So, both TRPV1 and TRPV2 receptors are activated by CBD [126, 127], allowing an increase of intracellular Ca^2+^ [128]. The activation and the subsequent desensitization of these receptor proteins, which are involved in transducing acute inflammatory and chronic pain (especially TRPV1), might be responsible for the antihyperalgesic actions of CBD [127]. Interestingly, the results found on prostate and skin cancers cells have shown that both TRPV1 and TRPV2 are involved in cancer progression, thanks to their ability to interact with G-proteins and, therefore, to interfere with intracellular signaling and to modulate intracellular Ca2+ [129, 130]. Protein receptors are differently up- and downregulated in cancer tissues, thus indicating a possible different role in cancer progression. In particular, TRPV1 is upregulated in glioma, prostate, and pancreas cancers, whereas it is downregulated in hepatocellular carcinoma (HCC), bladder, and skin cancer [123]. TRPV2 is upregulated in bladder, prostate, and HCC, while it is downregulated in glioma cancer cells [123]. In this context, it has been demonstrated that the concomitant overexpression of TRPV2 and insulin-like growth factor 1 (IGF-1) suggests that TRPV2 might control the urothelial cancer cell growth and progression through the modulation of IGF-1 pathway [131]. In U87MG glioblastoma/astrocytoma cell line, TRPV2 decreases cell malignancy and cell survival in an ERK dependent manner [123]. In addition, TRPV1 has been found to be colocalized with the proapoptotic protein Fas/CD95, and, when stimulated with the agonist capsaicin, it causes a cell cycle arrest in G0/G1 in RT4 and apoptosis in urothelial cancer cells [132]. So, the interaction with an agonist on TRPV1 or TRPV2 receptors could originate different biological responses, depending on the distribution of TRPV, together with the fine interactions with other molecular complexes. In this view, CBD has been found to inhibit the multidrug resistance (MDR), by interacting with TRPV1 and CB_2_ at the same time (Figure 3). Indeed, in the MDR CEM/VLB100 cell model, Arnold et al. have reported that the treatment with CBD caused a downregulation of P-glycoprotein (P-gp) expression and an increase of the cytotoxic effects of vinblastine, whose P-gp is the substrate [133] However, this effect was mediated both by the cooperation of CBD with TRPV1 and by CB_2_ receptors, indicating once more the intricate complexity of interaction between biological pathways. Moreover, the ability of CBD to increase the cytotoxic activity of anticancer agents, such as temozolomide, doxorubicin, and carmustine in U87MG cancer cells, allowed Nabissi et al. [134] to discover that this effect was due to the interaction of CBD with TRPV2 receptor, which resulted in an increase of drug uptake. This interesting finding could be of relevance also in the management of glioma cancer stem cells (GSCs). Indeed, it has been reported that TRPV2 activation led to a GSCs differentiation and, therefore, to an inhibition of their proliferation [135]. This effect could be due, at least in part, to the ability of CBD to upregulate the prodifferentiation factor ID2 and to downregulate the metastatic factor ID1 [136], since both these proteins play an important role in spreading neuroblastoma cells [137]. Taking into account the fact that GSCs are the major factor responsible for glioma recurrence, the use of CBD could also be a valuable tool against the proliferation of the GSCs subpopulations present in glioma/glioblastoma cancers.

## 6. Hemp Extracts and CBD between Present and Future

This review is mainly focused on the role of CBD and related nonpsychoactive compounds in the modulation of the inflammatory processes linked to the degenerative diseases and, in particular, to cancer. From a pharmaceutical point of view, CBD represents at the moment the most promising compound present in* C. sativa*. Although this component is well-known mainly for its antioxidant and anti-inflammatory activities, a number of researches pointed out its ability to interfere with cell proliferation apoptosis and cancer growth. If we consider also that cancer biology and inflammation share several common pathways in some stages of their biological processes, CBD might be a potential important tool in the control of cancer spread and growth.

It is important however to consider also other issues regarding cannabinoids and their use, comprising the poor availability of the plant material, the uncertainties on the quality of the products, and the safety of CBD. For these reasons, CBD is under scrutiny at many levels, ranging from national health organizations to FDA and WHO. Up to now, many clinical trials have been performed on Sativex®, which is a combination of Δ^9^-THC and CBD, or on Epidiolex®, which is currently in phase three, with encouraging results against a severe form of epilepsy in children.

However, one of the main points under debate is whether cannabinoids and CBD, in particular, are safe for consumers at the doses found to be active in the experimental conditions, by taking into account the fact that there is only limited knowledge about the long-term effects of chronic use and drug-drug interactions between CBD and other medications, although human studies have indicated that CBD is very well tolerated even at high doses. Another important issue is whether or not* Cannabis* extracts or CBD are simply a food supplement, a pharmaceutical product, or other. If on one hand this perplexity is justified by the need for a reliable evaluation of the balance between efficacy and side effects, on the other hand it must be recognized that, in some cases, an unconscious prejudice seems to hover on* C. sativa, *mainly because of its history of drug of abuse.

We believe that although an important number of studies regarding the therapeutic effects of CBD have been performed in the last decade, there is no solid clinical evidence yet to support that cannabinoids can effectively and safely treat cancer in humans. However, by taking into account the fact that hemp extracts with low Δ^9^-THC concentration but rich in nonpsychoactive compounds are still poorly studied from a pharmacological and molecular point of view, we think that they could be a precious resource for future treatment of both acute and chronic diseases. In addition, by considering the availability of specific cultivars containing different amounts of active compounds, such as flavonoids and terpenes, it might be possible to select the appropriate variety enriched of a specific class of compounds to be used for a specific disease. Moreover, if we consider that the treatment of most degenerative diseases is still far from achieving full success, the research on hemp and CBD extracts is strongly encouraged in order to have enough data for a rational clinical application.

## References

[1] Hartsel J. A., Eades J., Hickory B., Makriyannis A. (2016). Cannabis sativa and Hemp. *Nutraceuticals: Efficacy, Safety and Toxicity*.

[2] Andre C. M., Hausman J., Guerriero G. (2016). Cannabis sativa: The Plant of the Thousand and One Molecules. *Frontiers in Plant Science*.

[3] Appendino G., Chianese G., Taglialatela-Scafati O. (2011). Cannabinoids: Occurrence and medicinal chemistry. *Current Medicinal Chemistry*.

[4] Thomas B. F., ElSohly M. A. (2015). The analytical chemistry of *Cannabis*: quality assessment, assurance and regulation of medicinal marijuana and cannabinoid preparations.

[5] European Commission, Food safety, plant variety catalogues, databases and information systems. https://ec.europa.eu/food/plant/plant_propagation_material/plant_variety_catalogues_databases_en.

[6] Hanuš L. O., Meyer S. M., Muñoz E., Taglialatela-Scafati O., Appendino G. (2016). Phytocannabinoids: A unified critical inventory. *Natural Product Reports*.

[7] Brighenti V., Pellati F., Steinbach M., Maran D., Benvenuti S. (2017). Development of a new extraction technique and HPLC method for the analysis of non-psychoactive cannabinoids in fibre-type Cannabis sativa L. (hemp). *Journal of Pharmaceutical and Biomedical Analysis*.

[8] Pellati F., Brighenti V., Sperlea J., Marchetti L., Bertelli D., Benvenuti S. (2018). New Methods for the Comprehensive Analysis of Bioactive Compounds in Cannabis sativa L. (hemp). *Molecules*.

[9] ElSohly M. A., Slade D. (2005). Chemical constituents of marijuana: the complex mixture of natural cannabinoids. *Life Sciences*.

[10] De Backer B., Debrus B., Lebrun P. (2009). Innovative development and validation of an HPLC/DAD method for the qualitative and quantitative determination of major cannabinoids in cannabis plant material. *Journal of Chromatography B*.

[11] Izzo A. A., Borrelli F., Capasso R., Di Marzo V., Mechoulam R. (2009). Non-psychotropic plant cannabinoids: new therapeutic opportunities from an ancient herb. *Trends in Pharmacological Sciences*.

[12] Fernández-Ruiz J., Moreno-Martet M., Rodríguez-Cueto C. (2011). Prospects for cannabinoid therapies in basal ganglia disorders. *British Journal of Pharmacology*.

[13] Alexander S. P. H. (2016). Therapeutic potential of cannabis-related drugs. *Progress in Neuro-Psychopharmacology & Biological Psychiatry*.

[14] Campos A. C., Fogaça M. V., Sonego A. B., Guimarães F. S. (2016). Cannabidiol, neuroprotection and neuropsychiatric disorders. *Pharmacological Research*.

[15] Brenneisen R., ElSohly M. A. (2007). Chemistry and analysis of phytocannabinoids and other Cannabis constituents. *Marijuana and the Cannabinoids*.

[16] Pisanti S., Malfitano A. M., Ciaglia E. (2017). Cannabidiol: State of the art and new challenges for therapeutic applications. *Pharmacology & Therapeutics*.

[17] Pollastro F., Minassi A., Fresu L. G. (2018). Cannabis Phenolics and their Bioactivities. *Current Medicinal Chemistry*.

[18] Werz O., Seegers J., Schaible A. M. (2014). Cannflavins from hemp sprouts, a novel cannabinoid-free hemp food product, target microsomal prostaglandin E2 synthase-1 and 5-lipoxygenase. *PharmaNutrition*.

[19] Allegrone G., Pollastro F., Magagnini G. (2017). The Bibenzyl Canniprene Inhibits the Production of Pro-Inflammatory Eicosanoids and Selectively Accumulates in Some Cannabis sativa Strains. *Journal of Natural Products*.

[20] Grivennikov S. I., Greten F. R., Karin M. (2010). Immunity, Inflammation, and Cancer. *Cell*.

[21] Namdar D., Koltai H. (2018). Medical Cannabis for the treatment of inflammation. *Nat. Prod. Commun*.

[22] Mackie K., Stella N. (2006). Cannabinoid receptors and endocannabinoids: evidence for new players. *The AAPS Journal*.

[23] Jean-Gilles L., Braitch M., Latif M. L. (2015). Effects of pro-inflammatory cytokines on cannabinoid CB1 and CB2 receptors in immune cells. *Acta Physiol*.

[24] Fijal K., Filip M. (2016). Clinical/therapeutic approaches for cannabinoid ligands in central and peripheral nervous system diseases: Mini review. *Clinical Neuropharmacology*.

[25] Mccoy K. L. (2016). Interaction between Cannabinoid System and Toll-Like Receptors Controls Inflammation. *Mediators of Inflammation*.

[26] Nagarkatti P., Pandey R., Rieder S. A., Hegde V. L., Nagarkatti M. (2009). Cannabinoids as novel anti-inflammatory drugs. *Future Medicinal Chemistry*.

[27] Burstein S. (2015). Cannabidiol (CBD) and its analogs: A review of their effects on inflammation. *Bioorganic & Medicinal Chemistry*.

[28] Jamontt J. M., Molleman A., Pertwee R. G., Parsons M. E. (2010). The effects of Δ9-tetrahydrocannabinol and cannabidiol alone and in combination on damage, inflammation and in vitro motility disturbances in rat colitis. *British Journal of Pharmacology*.

[29] de Filippis D., Esposito G., Cirillo C. (2011). Cannabidiol reduces intestinal inflammation through the control of neuroimmune axis. *PLoS ONE*.

[30] Sido J. M., Jackson A. R., Nagarkatti P. S., Nagarkatti M. (2016). Marijuana-derived Δ-9-tetrahydrocannabinol suppresses Th1/Th17 cell-mediated delayed-type hypersensitivity through microRNA regulation. *Journal of Molecular Medicine*.

[31] Berdyshev E., Boichot E., Corbel M., Germain N., Lagente V. (1998). Effects of cannabinoid receptor ligands on LPS-induced pulmonary inflammation in mice. *Life Sciences*.

[32] Roth M. D., Castaneda J. T., Kiertscher S. M. (2015). Exposure to Δ9-tetrahydrocannabinol impairs the differentiation of human monocyte-derived dendritic cells and their capacity for T cell activation. *Journal of Neuroimmune Pharmacology*.

[33] Ngaotepprutaram T., Kaplan B. L., Kaminski N. E. (2013). Impaired NFAT and NF*κ*B activation are involved in suppression of CD40 ligand expression by Δ9-tetrahydrocannabinol in human CD4+ T cells. *Toxicology and Applied Pharmacology*.

[34] Chang Y.-H., Lee S. T., Lin W.-W. (2001). Effects of cannabinoids on LPS-stimulated inflammatory mediator release from macrophages: involvement of eicosanoids. *Journal of Cellular Biochemistry*.

[35] Ruhaak L. R., Felth J., Karlsson P. C., Rafter J. J., Verpoorte R., Bohlin L. (2011). Evaluation of the cyclooxygenase inhibiting effects of six major cannabinoids isolated from Cannabis sativa. *Biological & Pharmaceutical Bulletin*.

[36] Shivers S. C., Newton C., Friedman H., Klein T. W. (1994). Δ9-tetrahydrocannabinol (THC) modulates IL-1 bioactivity in human monocyte/macrophage cell lines. *Life Sciences*.

[37] Lombard C., Nagarkatti M., Nagarkatti P. S. (2005). Targeting cannabinoid receptors to treat leukemia: Role of cross-talk between extrinsic and intrinsic pathways in Δ9- tetrahydrocannabinol (THC)-induced apoptosis of Jurkat cells. *Leukemia Research*.

[38] Jia W., Hegde V. L., Singh N. P. (2006). Δ9-tetrahydrocannabinol-induced apoptosis in Jurkat leukemia T cells is regulated by translocation of bad to mitochondria. *Molecular Cancer Research*.

[39] Costa B., Colleoni M., Conti S. (2004). Oral anti-inflammatory activity of cannabidiol, a non-psychoactive constituent of cannabis, in acute carrageenan-induced inflammation in the rat paw. *Naunyn-Schmiedeberg's Archives of Pharmacology*.

[40] Takeda S., Okazaki H., Ikeda E. (2014). Down-regulation of cyclooxygenase-2 (cox-2) by cannabidiolic acid in human breast cancer cells. *Journal of Toxicological Sciences*.

[41] Ben-Shabat S., Hanuš L. O., Katzavian G., Gallily R. (2006). New cannabidiol derivatives: Synthesis, binding to cannabinoid receptor, and evaluation of their antiinflammatory activity. *Journal of Medicinal Chemistry*.

[42] Watzl B., Scuderi P., Watson R. R. (1991). Marijuana components stimulate human peripheral blood mononuclear cell secretion of interferon-gamma and suppress interleukin-1 alpha in vitro. *International Journal of Immunopharmacology*.

[43] Petrosino S., Verde R., Vaia M., Allarà M., Iuvone T., Di Marzo V. (2018). Anti-inflammatory Properties of Cannabidiol, a Nonpsychotropic Cannabinoid, in Experimental Allergic Contact Dermatitis. *The Journal of Pharmacology and Experimental Therapeutics*.

[44] Borrelli F., Fasolino I., Romano B. (2013). Beneficial effect of the non-psychotropic plant cannabinoid cannabigerol on experimental inflammatory bowel disease. *Biochemical Pharmacology*.

[45] Izzo A. A., Capasso R., Aviello G. (2012). Inhibitory effect of cannabichromene, a major non-psychotropic cannabinoid extracted from Cannabis sativa, on inflammation-induced hypermotility in mice. *British Journal of Pharmacology*.

[46] Barrett M. L., Gordon D., Evans F. J. (1985). Isolation from cannabis sativa L. of cannflavin-a novel inhibitor of prostaglandin production. *Biochemical Pharmacology*.

[47] Yoon W.-J., Lee N. H., Hyun C.-G. (2010). Limonene suppresses lipopolysaccharide-induced production of nitric oxide, prostaglandin E2, and pro-inflammatory cytokines in RAW 264.7 macrophages. *Journal of Oleo Science*.

[48] Gertsch J., Leonti M., Raduner S. (2008). Beta-caryophyllene is a dietary cannabinoid. *Proceedings of the National Acadamy of Sciences of the United States of America*.

[49] DiPatrizio N. V. (2016). Endocannabinoids in the Gut. *Cannabis and Cannabinoid Research*.

[50] Alhamoruni A., Lee A. C., Wright K. L., Larvin M., O'Sullivan S. E. (2010). Pharmacological effects of cannabinoids on the Caco-2 cell culture model of intestinal permeability. *The Journal of Pharmacology and Experimental Therapeutics*.

[51] Alhamoruni A., Wright K. L., Larvin M., O'Sullivan S. E. (2012). Cannabinoids mediate opposing effects on inflammation-induced intestinal permeability. *British Journal of Pharmacology*.

[52] Couch D. G., Tasker C., Theophilidou E., Lund J. N., O'Sullivan S. E. (2017). Cannabidiol and palmitoylethanolamide are anti-inflammatory in the acutely inflamed human colon. *Clinical Science*.

[53] Gigli S., Seguella L., Pesce M. (2017). Cannabidiol restores intestinal barrier dysfunction and inhibits the apoptotic process induced by Clostridium difficile toxin A in Caco-2 cells. *United European Gastroenterology Journal*.

[54] Kalaydina R.-V., Qorri B., Szewczuk M. R. (2017). Preventing negative shifts in gut microbiota with Cannabis therapy: implications for colorectal cancer. *Adv. Res. Gastroentero. Hepatol*.

[55] Mackie K., Lai Y., Westenbroek R., Mitchell R. (1995). Cannabinoids activate an inwardly rectifying potassium conductance and inhibit Q-type calcium currents in AtT20 cells transfected with rat brain cannabinoid receptor. *The Journal of Neuroscience*.

[56] Maresz K., Pryce G., Ponomarev E. D. (2007). Direct suppression of CNS autoimmune inflammation via the cannabinoid receptor CB1 on neurons and CB2 on autoreactive T cells. *Nature Medicine*.

[57] Klein T. W., Lane B., Newton C. A., Friedman H. (2000). The cannabinoid system and cytokine network. *Proceedings of the Society for Experimental Biology and Medicine*.

[58] Thors L., Bergh A., Persson E. (2010). Fatty acid amide hydrolase in prostate cancer: Association with disease severity and outcome, CB1 receptor expression and regulation by IL-4. *PLoS ONE*.

[59] Maccarrone M., Valensise H., Bari M., Lazzarin N., Romanini C., Finazzi-Agrò A. (2001). Progesterone up-regulates anandamide hydrolase in human lymphocytes: Role of cytokines and implications for fertility. *The Journal of Immunology*.

[60] Rubio-Araiz A., Arévalo-Martín Á., Gómez-Torres O. (2008). The endocannabinoid system modulates a transient TNF pathway that induces neural stem cell proliferation. *Molecular and Cellular Neuroscience*.

[61] Rajesh M., Mukhopadhyay P., Bátkai S. (2007). CB2-receptor stimulation attenuates TNF-*α*-induced human endothelial cell activation, transendothelial migration of monocytes, and monocyte-endothelial adhesion. *American Journal of Physiology-Heart and Circulatory Physiology*.

[62] Vivekanantham S., Shah S., Dewji R., Dewji A., Khatri C., Ologunde R. (2015). Neuroinflammation in Parkinson's disease: Role in neurodegeneration and tissue repair. *International Journal of Neuroscience*.

[63] Koudriavtseva T., Mainero C. (2016). Neuroinflammation, neurodegeneration and regeneration in multiple sclerosis: Intercorrelated manifestations of the immune response. *Neural Regeneration Research*.

[64] Gordon R., Woodruff T. M., Baekelandt V., Lobbestael E. (2017). Neuroinflammation as a therapeutic target in neurodegenerative diseases. *Disease-modifying targets in neurodegenerative disorders*.

[65] Heneka M. T., Carson M. J., Khoury J. El. (2015). Neuroinflammation in Alzheimer's disease. *The Lancet Neurology*.

[66] Gelders Géraldine, Baekelandt Veerle, Van der Perren Anke (2018). Linking Neuroinflammation and Neurodegeneration in Parkinson’s Disease. *Journal of Immunology Research*.

[67] Hirsch E. C., Vyas S., Hunot S. (2012). Neuroinflammation in Parkinson's disease. *Parkinsonism & Related Disorders*.

[68] Chitnis T. (2007). The Role of CD4 T Cells in the Pathogenesis of Multiple Sclerosis. *International Review of Neurobiology*.

[69] Kaskow B. J., Baecher-Allan C. (2018). Effector T Cells in Multiple Sclerosis. *Cold Spring Harbor Perspectives in Medicine*.

[70] Borgonetti V., Governa P., Montopoli M., Biagi M. (2018). Cannabis sativa L. constituents and their role in neuroinflammation. *Curr. Bioact. Compd*.

[71] Janefjord E., Mååg J. L. V., Harvey B. S., Smid S. D. (2014). Cannabinoid effects on *β* amyloid fibril and aggregate formation, neuronal and microglial-activated neurotoxicity in vitro. *Cellular and Molecular Neurobiology*.

[72] Kozela E., Pietr M., Juknat A., Rimmerman N., Levy R., Vogel Z. (2010). Cannabinoids delta(9)-tetrahydrocannabinol and cannabidiol differentially inhibit the lipopolysaccharide-activated NF-kappaB and interferon-beta/STAT proinflammatory pathways in BV-2 microglial cells. *The Journal of Biological Chemistry*.

[73] Hunter S. A., Burstein S. H. (1997). Receptor mediation in cannabinoid stimulated arachidonic acid mobilization and anandamide synthesis. *Life Sciences*.

[74] Solinas M., Massi P., Cinquina V. (2013). Cannabidiol, a Non-Psychoactive Cannabinoid Compound, Inhibits Proliferation and Invasion in U87-MG and T98G Glioma Cells through a Multitarget Effect. *PLoS ONE*.

[75] Martín-Moreno A. M., Reigada D., Ramírez B. G. (2011). Cannabidiol and other cannabinoids reduce microglial activation in vitro and in vivo: Relevance to alzheimer's disease. *Molecular Pharmacology*.

[76] Esposito G., Scuderi C., Valenza M. (2011). Cannabidiol reduces A*β*-induced neuroinflammation and promotes hippocampal neurogenesis through PPAR*γ* involvement. *PLoS ONE*.

[77] Dirikoc S., Priola S. A., Marella M., Zsürger N., Chabry J. (2007). Nonpsychoactive cannabidiol prevents prion accumulation and protects neurons against prion toxicity. *The Journal of Neuroscience*.

[78] Corsi L., Pellati F., Brighenti V., Plessi N., Benvenuti S. (2018). Chemical composition and in vitro neuroprotective activity of fibre-type Cannabis sativa L. (hemp). *Current Bioactive Compounds*.

[79] Gugliandolo A., Pollastro F., Grassi G., Bramanti P., Mazzon E. (2018). In Vitro Model of Neuroinflammation: Efficacy of Cannabigerol, a Non-Psychoactive Cannabinoid. *International Journal of Molecular Sciences*.

[80] Guo K., Mou X., Huang J., Xiong N., Li H. (2014). Trans-caryophyllene suppresses hypoxia-induced neuroinflammatory responses by inhibiting NF-*κ*B activation in microglia. *Journal of Molecular Neuroscience*.

[81] Watt G., Karl T. (2017). In vivo evidence for therapeutic properties of cannabidiol (CBD) for Alzheimers Disease. *Front. Pharmacol*.

[82] Scuderi C., Steardo L., Esposito G. (2014). Cannabidiol promotes amyloid precursor protein ubiquitination and reduction of beta amyloid expression in SHSY5YAPP+ cells through PPAR*γ* involvement. *Phytotherapy Research*.

[83] Hughes B., Herron C. E. (2018). Cannabidiol Reverses Deficits in Hippocampal LTP in a Model of Alzheimer’s Disease. *Neurochemical Research*.

[84] Kundu K., Surh Y. J. (2008). Inflammation: gearing the journey to cancer. *Mutat. Res*.

[85] Mantovani A., Allavena P., Sica A., Balkwill F. (2008). Cancer-related inflammation. *Nature*.

[86] Dvorak H. F. (1986). Tumors: wounds that do not heal: similarities between tumor stroma generation and wound healing. *The New England Journal of Medicine*.

[87] Crusz S. M., Balkwill F. R. (2015). Inflammation and cancer: advances and new agents. *Nature Reviews Clinical Oncology*.

[88] Grivennikov S. I., Karin M. (2010). Inflammation and oncogenesis: a vicious connection. *Curr. Opin. Genet. Dev*.

[89] Tanaka K., Babic I., Nathanson D. (2011). Oncogenic EGFR signaling activates an mTORC2-NF-*κ*B pathway that promotes chemotherapy resistance. *Cancer Discovery*.

[90] Berasain C., Perugorria M. J., Latasa M. U. (2009). The Epidermal Growth Factor Receptor: A Link Between Inflammation and Liver Cancer. *Experimental Biology and Medicine*.

[91] Elbaz M., Nasser M. W., Ravi J. (2015). Modulation of the tumor microenvironment and inhibition of EGF/EGFR pathway: Novel anti-tumor mechanisms of Cannabidiol in breast cancer. *Molecular Oncology*.

[92] Chiurchiù V., Lanuti M., De Bardi M., Battistini L., Maccarrone M. (2015). The differential characterization of GPR55 receptor in human peripheral blood reveals a distinctive expression in monocytes and NK cells and a proinflammatory role in these innate cells. *International Immunology*.

[93] Wang D., Wang H., Ning W., Backlund M. G., Dey S. K., DuBois R. N. (2008). Loss of cannabinoid receptor 1 accelerates intestinal tumor growth. *Cancer Research*.

[94] Mukhopadhyay B., Schuebel K., Mukhopadhyay P. (2015). Cannabinoid receptor 1 promotes hepatocellular carcinoma initiation and progression through multiple mechanisms. *Hepatology*.

[95] Benz A. H., Renné C., Maronde E. (2013). Expression and functional relevance of cannabinoid receptor 1 in hodgkin lymphoma. *PLoS ONE*.

[96] Messalli E. M., Grauso F., Luise R., Angelini A., Rossiello R. (2014). Cannabinoid receptor type 1 immunoreactivity and disease severity in human epithelial ovarian tumors. *American Journal of Obstetrics & Gynecology*.

[97] Pérez-Gómez E., Andradas C., Blasco-Benito S. (2015). Role of Cannabinoid Receptor CB2 in HER2 Pro-oncogenic Signaling in Breast Cancer. *JNCI: Journal of the National Cancer Institute*.

[98] Dumitru C. A., Sandalcioglu I. E., Karsak M. (2018). Cannabinoids in Glioblastoma Therapy: New Applications for Old Drugs. *Frontiers in Molecular Neuroscience*.

[99] Jung C. K., Kang W. K., Park J. M. (2013). Expression of the cannabinoid type I receptor and prognosis following surgery in colorectal cancer. *Oncology Letters*.

[100] Engels F. K., de Jong F. A., Mathijssen R. H. J., Erkens J. A., Herings R. M., Verweij J. (2007). Medicinal cannabis in oncology. *European Journal of Cancer*.

[101] Munson A. E., Harris L. S., Friedman M. A., Dewey W. L., Carchman R. A. (1975). Antineoplastic Activity of Cannabinoids2. *JNCI: Journal of the National Cancer Institute*.

[102] Galve-Roperh I., Sánchez C., Cortés M. L., Del Pulgar T. G., Izquierdo M., Guzmán M. (2000). Anti-tumoral action of cannabinoids: Involvement of sustained ceramide accumulation and extracellular signal-regulated kinase activation. *Nature Medicine*.

[103] Sánchez C., de Ceballos M. L., Gomez del Pulgar T. (2001). Inhibition of glioma growth in vivo by selective activation of the CB2 cannabinoid receptor. *Cancer Res*.

[104] Casanova M. L., Blázquez C., Martínez-Palacio J. (2003). Inhibition of skin tumor growth and angiogenesis in vivo by activation of cannabinoid receptors. *The Journal of Clinical Investigation*.

[105] Blázquez C., Carracedo A., Barrado L. (2006). Cannabinoid receptors as novel targets for the treatment of melanoma.. *The FASEB journal : official publication of the Federation of American Societies for Experimental Biology*.

[106] Carracedo A., Gironella M., Lorente M. (2006). Cannabinoids induce apoptosis of pancreatic tumor cells via endoplasmic reticulum stress-related genes. *Cancer Research*.

[107] Cianchi F., Papucci L., Schiavone N. (2008). Cannabinoid receptor activation induces apoptosis through tumor necrosis factor alpha-mediated ceramide de novo synthesis in colon cancer cells. *Clinical Cancer Research*.

[108] Bifulco M., Di Marzo V. (2002). Targeting the endocannabinoid system in cancer therapy: A call for further research. *Nature Medicine*.

[109] Bifulco M., Laezza C., Pisanti S., Gazzerro P. (2006). Cannabinoids and cancer: Pros and cons of an antitumour strategy. *British Journal of Pharmacology*.

[110] Freimuth N., Ramer R., Hinz B. (2010). Antitumorigenic effects of cannabinoids beyond apoptosis. *The Journal of Pharmacology and Experimental Therapeutics*.

[111] Sawzdargo M., Nguyen T., Lee D. K. (1999). Identification and cloning of three novel human G protein-coupled receptor genes GPR52, ΨGPR53 and GPR55: GPR55 is extensively expressed in human brain. *Brain Research*.

[112] Fredriksson R., Lagerström M. C., Lundin L.-G., Schiöth H. B. (2003). The G-protein-coupled receptors in the human genome form five main families. Phylogenetic analysis, paralogon groups, and fingerprints. *Molecular Pharmacology*.

[113] Leyva-Illades D., DeMorrow S. (2013). Orphan G protein receptor GPR55 as an emerging target in cancer therapy and management. *Cancer Management and Research*.

[114] Henstridge C. M., Balenga N. A., Schröder R. (2010). GPR55 ligands promote receptor coupling to multiple signalling pathways. *British Journal of Pharmacology*.

[115] Lauckner J. E., Jensen J. B., Chen H.-Y., Lu H.-C., Hille B., Mackie K. (2008). GPR55 is a cannabinoid receptor that increases intracellular calcium and inhibits M current. *Proceedings of the National Acadamy of Sciences of the United States of America*.

[116] Oka S., Kimura S., Toshida T., Ota R., Yamashita A., Sugiura T. (2010). Lysophosphatidylinositol induces rapid phosphorylation of p38 mitogen-activated protein kinase and activating transcription factor 2 in HEK293 cells expressing GPR55 and IM-9 lymphoblastoid cells. *The Journal of Biochemistry*.

[117] Andradas C., Caffarel M. M., Pérez-Gómez E. (2011). The orphan G protein-coupled receptor GPR55 promotes cancer cell proliferation via ERK. *Oncogene*.

[118] Pérez-Gómez E., Andradas C., Flores J. M. (2013). The orphan receptor GPR55 drives skin carcinogenesis and is upregulated in human squamous cell carcinomas. *Oncogene*.

[119] Ryberg E., Larsson N., Sjögren S. (2007). The orphan receptor GPR55 is a novel cannabinoid receptor. *British Journal of Pharmacology*.

[120] Kapur A., Zhao P., Sharir H. (2009). Atypical responsiveness of the orphan receptor GPR55 to cannabinoid ligands. *The Journal of Biological Chemistry*.

[121] Shrivastava A., Kuzontkoski P. M., Groopman J. E., Prasad A. (2011). Cannabidiol Induces Programmed Cell Death in Breast Cancer Cells by Coordinating the Cross-talk between Apoptosis and Autophagy. *Molecular Cancer Therapeutics*.

[122] Zhang X., Maor Y., Wang J. F., Kunos G., Groopman J. E. (2010). Endocannabinoid-like N-arachidonoyl serine is a novel pro-angiogenic mediator. *British Journal of Pharmacology*.

[123] Santoni G., Farfariello V., Amantini C. (2011). TRPV Channels in Tumor Growth and Progression. *Transient Receptor Potential Channels*.

[124] Vennekens R., Owsianik G., Nilius B. (2008). Vanilloid transient receptor potential cation channels: An overview. *Current Pharmaceutical Design*.

[125] Perálvarez-Marín A., Doñate-Macian P., Gaudet R. (2013). What do we know about the transient receptor potential vanilloid 2 (TRPV2) ion channel?. *FEBS Journal*.

[126] Bisogno T., Hanuš L., De Petrocellis L. (2001). Molecular targets for cannabidiol and its synthetic analogues: Effect on vanilloid VR1 receptors and on the cellular uptake and enzymatic hydrolysis of anandamide. *British Journal of Pharmacology*.

[127] Qin N., Neeper M. P., Liu Y., Hutchinson T. L., Lubin M. L., Flores C. M. (2008). TRPV2 is activated by cannabidiol and mediates CGRP release in cultured rat dorsal root *Ganglion neurons*. *The Journal of Neuroscience*.

[128] Ligresti A., Moriello A. S., Starowicz K. (2006). Antitumor activity of plant cannabinoids with emphasis on the effect of cannabidiol on human breast carcinoma. *The Journal of Pharmacology and Experimental Therapeutics*.

[129] Flourakis M., Prevarskaya N. (2009). Insights into Ca2+ homeostasis of advanced prostate cancer cells. *Biochimica et Biophysica Acta (BBA) - Molecular Cell Research*.

[130] Bode A. M., Cho Y.-Y., Zheng D. (2009). Transient receptor potential type vanilloid 1 suppresses skin carcinogenesis. *Cancer Research*.

[131] Rochester M. A., Patel N., Turney B. W. (2007). The type 1 insulin-like growth factor receptor is over-expressed in bladder cancer. *BJU International*.

[132] Amantini C., Ballarini P., Caprodossi S. (2009). Triggering of transient receptor potential vanilloid type 1 (TRPV1) by capsaicin induces Fas/CD95-mediated apoptosis of urothelial cancer cells in an ATM-dependent manner. *Carcinogenesis*.

[133] Arnold J. C., Hone P., Holland M. L., Allen J. D. (2012). CB2and TRPV1 receptors mediate cannabinoid actions on MDR1 expression in multidrug resistant cells. *Pharmacological Reports*.

[134] Nabissi M., Morelli M. B., Santoni M., Santoni G. (2013). Triggering of the TRPV2 channel by cannabidiol sensitizes glioblastoma cells to cytotoxic chemotherapeutic agents. *Carcinogenesis*.

[135] Morelli M. B., Nabissi M., Amantini C. (2012). The transient receptor potential vanilloid-2 cation channel impairs glioblastoma stem-like cell proliferation and promotes differentiation. *International Journal of Cancer*.

[136] McAllister S. D., Murase R., Christian R. T. (2011). Pathways mediating the effects of cannabidiol on the reduction of breast cancer cell proliferation, invasion, and metastasis. *Breast Cancer Research and Treatment*.

[137] Perk J., Iavarone A., Benezra R. (2005). Id family of helix-loop-helix proteins in cancer. *Nature Reviews Cancer*.

